# LKB1/p53/TIGAR/autophagy-dependent VEGF expression contributes to PM2.5-induced pulmonary inflammatory responses

**DOI:** 10.1038/s41598-019-53247-6

**Published:** 2019-11-12

**Authors:** Huan Xu, Xiuduan Xu, Hongli Wang, Aodeng Qimuge, Shasha Liu, Yuanlian Chen, Chongchong Zhang, Meiru Hu, Lun Song

**Affiliations:** 1 Institute of Military Cognitive and Brain Sciences, 27 Taiping Road, Beijing, 100850 People’s Republic of China; 2 Anhui Medical University, 81 Meishan Road, Hefei, 230032 People’s Republic of China; 3 Laboratory of Cellular and Molecular Immunology, School of Medicine, Henan University, 357 Ximen Road, Kaifeng, 475004 People’s Republic of China; 4 Department of New Drug Screening Center, China Pharmaceutical University, 24 Tongjiaxiang, Nanjing, 210009 People’s Republic of China; 5 Department of Pathology, School of Basic Medical Sciences, Lanzhou University, Tianshui South Road, Lanzhou, 730000 People’s Republic of China; 6 Guiin Medical University, 1 Zhiyuan Road, Guilin, 541100 P.R. China; 7 Present Address: School of Life Sciences, Tsinghua University, Beijing, China

**Keywords:** Macroautophagy, Stress signalling

## Abstract

One of the health hazards of PM2.5 exposure is to induce pulmonary inflammatory responses. In our previous study, we demonstrated that exposing both the immortalized and primary human bronchial epithelial cells to PM2.5 results in a significant upregulation of VEGF production, a typical signaling event to trigger chronic airway inflammation. Further investigations showed that PM2.5 exposure strongly induces ATR/CHK1/p53 cascade activation, leading to the induction of DRAM1-dependent autophagy to mediate VEGF expression by activating Src/STAT3 pathway. In the current study, we further revealed that TIGAR was another transcriptional target of p53 to trigger autophagy and VEGF upregulation in Beas-2B cells after PM2.5 exposure. Furthermore, LKB1, but not ATR and CHK1, played a critical role in mediating p53/TIGAR/autophagy/VEGF pathway activation also by linking to Src/STAT3 signaling cascade. Therefore, on combination of the previous report, we have identified both ATR/CHK1/p53/DRAM1- and LKB1/p53/TIGAR- dependent autophagy in mediating VEGF production in the bronchial epithelial cells under PM2.5 exposure. Moreover, the *in vivo* study further confirmed VEGF induction in the airway potentially contributed to the inflammatory responses in the pulmonary vascular endothelium of PM2.5-treated rats. Therefore, blocking VEGF expression or autophagy induction might be the valuable strategies to alleviating PM2.5-induced respiratory injuries.

## Introduction

Particulate matter (PM) 2.5 is regarded as the main component of air pollution that can cause pathological responses primarily in the respiratory and cardiovascular system^1,2^. After inhalation, PM2.5 can enter the respiratory tract and lungs, deposit in the deep parts of the respiratory tract and induce a pulmonary inflammatory response, which is evidenced by the upregulation of several pro-inflammatory cytokines and adhesion molecules in the airway, such as interleukin (IL) -1β, IL-6, IL-8, tumor necrosis factor (TNF) α, intercellular adhesion molecule (ICAM1) 1, etc^3^. Longer exposure to PM2.5 can further induce the movement of the above pro-inflammatory factors into the systemic circulation and then cause several adverse responses in the cardiovascular system^4,5^. Therefore, disclosing the mechanism involving in the airway inflammatory response may be helpful to develop effective strategies to alleviate the cardiopulmonary pathologies induced by PM2.5.

Autophagy is an evolutionarily conserved catabolic process for degrading the proteins and the cytoplasmic components under both steady state and stress conditions^6^. The close correlation of autophagy with the various respiratory pathologies (such as asthma, respiratory tract infection, chronic obstructive pulmonary disease, etc.) has been well-described in the numerous studies. According to these findings, abnormal elevation of pulmonary autophagy contributes largely to the airway mucus hyper-secretion, airway epithelial fibrosis, impairment of respiratory antiviral responses, and airway remodeling^7–10^. Therefore, development of therapeutics targeting to the upstream signaling events leading to autophagic pathway activation are regarded as a useful way for manipulating lung diseases.

p53 is one of the master regulators of autophagy, which exerts its role within the nucleus by transcription-dependent manner or within the cytoplasm by transcription-independent manner^11^. In our previous study, we demonstrate that exposing immortalized and primary human bronchial epithelial cells to PM2.5 induces p53 transactivation and autophagy, which leads to a significant upregulation of VEGF production, a typical signaling event in mediating chronic inflammation and vascular endothelial dysfunction in the lung. Further investigations show that lysosomal protein DRAM1 functioned as the downstream target of p53 to orchestrate the induction of autophagy, therefore expand on the former mechanism of p53/DRAM1-dependent autophagy in modulating cell apoptosis in response to genotoxic stress to the airway inflammatory responses induced by PM2.5. In addition, we also demonstrate that ATR and CHK1 functioned as the upstream protein kinases for inducing p53 cascade activation, leading to the induction of DRAM1-dependent autophagy to mediate VEGF expression^12^. Therefore, we have illustrated the novel mechanism of p53-dependent autophagy in mediating the airway inflammatory responses upon PM2.5 exposure.

In the current study, we further investigated the signaling events leading to p53-dependent autophagy and the subsequent VEGF production and their contribution to pulmonary inflammation under PM2.5 exposure. And we found that LKB1 played a critical role in inducing p53-dependent autophagy by promoting the expression of another p53 transcriptional target, TIGAR. Therefore, on combination of the previous report, we have identified both ATR/CHK1/p53/DRAM1- and LKB1/p53/TIGAR- dependent autophagy in mediating VEGF production in the bronchial epithelial cells and its contribution to the pulmonary inflammatory responses induced by PM2.5.

## Results

### Induction of autophagy-related transcriptional targets of p53 upon PM2.5 exposure

In our previous study, we demonstrated that PM2.5 exposure strongly induces VEGF expression in Beas-2B human bronchial epithelial cells, but the levels of other pro-inflammatory cytokines (IL-1β, IL-6, IL-8, TNFα) in the cell culture media did not show obvious differences before and after PM2.5 exposure. In the attempt to disclose the mechanism involving in VEGF induction, we observe that PM2.5 exposure induces ATR/CHK1/p53 cascade activation, leading to the induction of DRAM1-dependent autophagy to mediate VEGF transcription and secretion by activating Src/STAT3 pathway^12^. In the current study, we further investigated the signaling events leading to p53-dependent autophagy and VEGF production in the PM2.5-treated Beas-2B cells.

**Figure 1:**
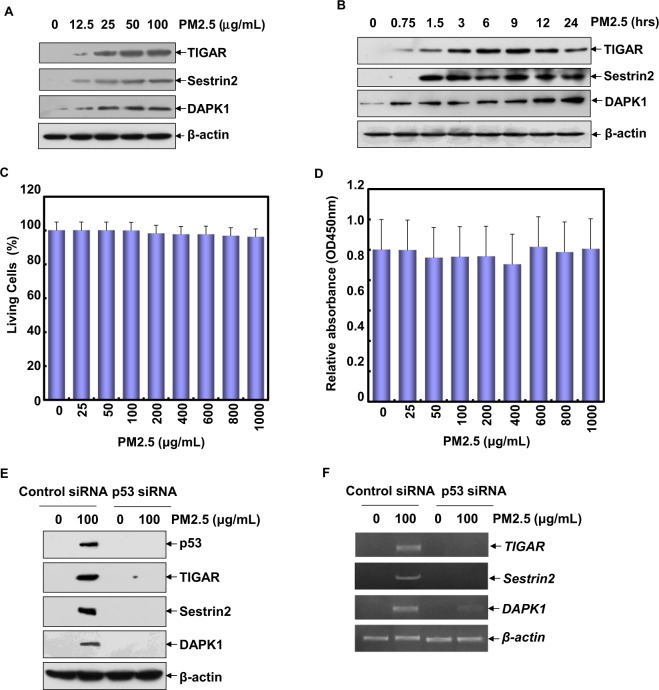
Induction of autophagy-related transcriptional targets of p53 upon PM2.5 exposure. (**A**) Beas-2B cells were treated with different doses of PM2.5 for 12 h and then the expression levels of TIGAR, Sestrin2 and DAPK1 were detected. (**B**) Beas-2B cells were treated with PM2.5 (100 μg/mL) for the indicated time periods and then the expression levels of TIGAR, Sestrin2 and DAPK1 were detected. (**C**,**D**) Beas-2B cells were treated with different doses of PM2.5 and then cell viability was determined by PI/Annexin V staining assay (**C**) and by using Cell Counting Kit (CCK-8) (**D**) at 24 h after PM2.5 exposure. (**E**) Beas-2B cells were transfected with p53 siRNA or the control siRNA and then treated with PM2.5 (100 μg/mL). The expression levels of p53, TIGAR, Sestrin2 and DAPK1 were detected at 12 h after PM2.5 exposure. (**F**) Beas-2B cells were transfected and treated as described in (**E**). Then the transcription of *TIGAR, Sestrin2 and DAPK1* were detected.

First, we tried to figure out more transcriptional targets of p53 that might be involved in mediating autophagy-dependent VEGF induction in Beas-2B cells upon PM2.5 exposure. Here we found that the expression levels of TIGAR, Sestrin2 and DAPK1, the autophagy-related p53 transcriptional targets^13–15^, were upregulated significantly in Beas-2B cells at 12 h after different doses of PM2.5 exposure (Fig. 1A) or upon a single dose of PM2.5 (100 μg/mL) exposure for the different time periods (Fig. 1B). Cell viability was not affected even under the exposure of 10 times the concentration of PM2.5 (1000 μg/mL) (Fig. 1C,D). Furthermore, knocking down the levels of p53 totally blocked the transcriptional induction and protein syntheses of these signaling molecules triggered by PM2.5 (Fig. 1E,F). These results indicate that TIGAR, Sestrin2 and DAPK1 function as the downstream transcriptional targets of p53 in the PM2.5-treated Beas-2B cells.

### TIGAR was involved in autophagy-dependent VEGF induction upon PM2.5 exposure

**Figure 2:**
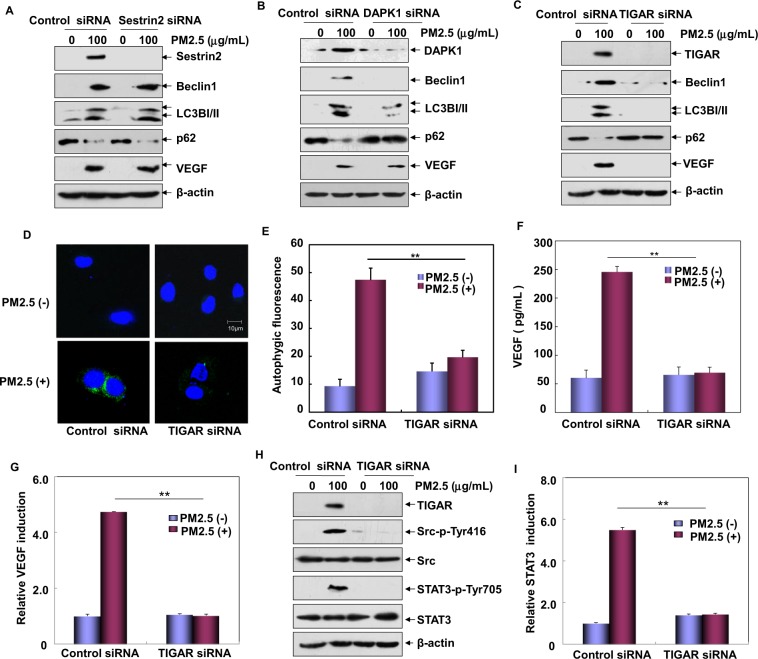
TIGAR was involved in autophagy-dependent VEGF induction upon PM2.5 exposure. (**A**–**C**) Beas-2B cells were transfected with TIGAR, Sestrin2, DAPK1 siRNA or their control siRNAs, respectively. Then the cells were exposed to PM2.5 (100 μg/mL) and the expression LC3BI/II, Beclin1, p62 and VEGF was examined at 12 h after PM2.5 exposure. (**D**,**E**) Beas-2B cells were transfected with TIGAR siRNA or control siRNA and then exposed to PM2.5 (100 μg/mL). Then autophagy was examined under confocal microscopy after the cells were stained with Cyto-ID Green Autophagy Detection Reagent (**D**) or by quantitative flow cytrometric assay (**E**) at 12 h after PM2.5 exposure. (**F**) Beas-2B cells were transfected as described in (**D**). Cell supernatants were collected and the expression of VEGF was examined by ELISA at 24 h after PM2.5 exposure (**P < 0.01). (**G**) Beas-2B cells stably transfected with *VEGF* promoter-driven luciferase reporter were transfected with TIGAR siRNA or control siRNA followed by treating with PM2.5 (100 μg/mL). Then, the induction of *VEGF* promoter-dependent luciferase activity was determined at 12 h after PM2.5 exposure (**P < 0.01). (**H**) Beas-2B cells were transfected and treated as described in (**D**). Then the activation of Src/STAT3 pathway was examined at 12 h after PM2.5 exposure. (**I**) Beas-2B cells stably transfected with the STAT3-dependent luciferase reporter were transfected with TIGAR siRNA or control siRNA followed by treating with PM2.5 (100 μg/mL). The induction of the STAT3-dependent luciferase activity was determined at 12 h after PM2.5 exposure (**P < 0.01).

Next, we investigated which downstream target(s) of p53 is/are involved in regulating autophagy-dependent VEGF expression under PM2.5 exposure. To this end, the expression of these p53 targets was inhibited by transfection with their respective siRNA in Beas-2B cells. Then we observed that inhibiting Sestrin2 expression neither affected the hallmarks level of autophagosome accumulation (LC3BII/I and Beclin1 upregulation) and autophagic degradation (p62 downregulation) nor altered upregulation of VEGF expression induced by PM2.5 (Fig. 2A). Interestingly, although abrogating DAPK1 expression efficiently inhibited the accumulation of LC3BI/II and Beclin1 and degradation of p62, upregulation of VEGF did not changed obviously under the same conditions (Fig. 2B). These results thus exclude the possible contribution of Sestrin2 and DAPK1 to the autophagy-dependent VEGF induction by PM2.5. Then, we found that inhibiting TIGAR expression not only blocked the upregulation of LC3BI/II and Beclin1 expression but also efficiently rescued the degradation of p62 upon PM2.5 exposure (Fig. 2C). Under the same conditions, the specific autophagic fluorescence signals from the Cyto-ID Autophagy Detection Reagent-stained Beas-2B cells were significantly decreased by knocking down TIGAR expression (Fig. 2D,E). These results indicate that TIGAR is another transcriptional target of p53 to trigger autophagy under PM2.5 exposure.

Next, we investigated the possible role of TIGAR in VEGF induction in response to PM2.5 exposure. Here we found that protein syntheses and secretion of VEGF were dramatically inhibited with the impairment of TIGAR expression (Fig. 2C,F). To further confirm these results, we next detected whether the transcription of *VEGF* is also regulated by TIGAR. Data in Fig. 2G showed that knocking down TIGAR expression significantly inhibited the induction of *VEGF* promoter-driven luciferase reporter activity upon PM2.5 exposure. These results together indicate the critical role of TIGAR in mediating the transcription and protein syntheses of VEGF induced by PM2.5.

According to our previous report, PM2.5-induced autophagy contributed to VEGF production by activating the Src/STAT3 pathway^12^. In the following study, we further observed that inhibiting TIGAR expression completely blocked that activation of Src protein kinase and the phosphorylation and transactivation of STAT3 (Fig. 2H,I), indicating that TIGAR also regulates VEGF expression though Src/STAT3 pathway-dependent manner.

### LKB1 is required for p53/TIGAR pathway activation in Beas-2B cells upon PM2.5 exposure

**Figure 3:**
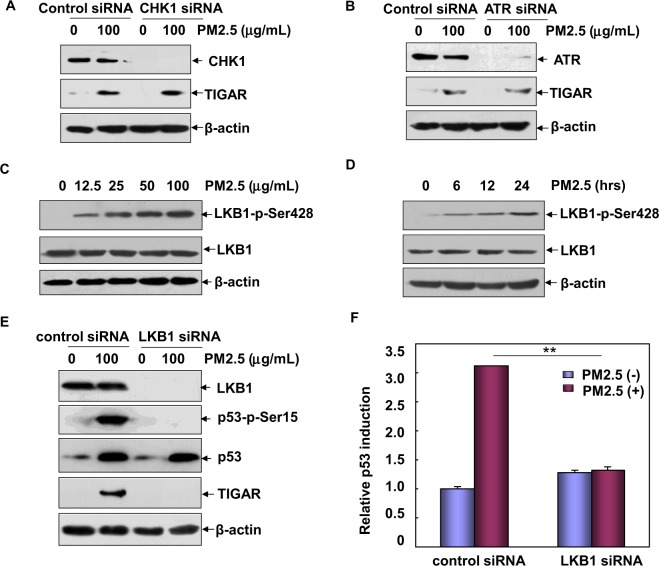
LKB1 is required for p53/TIGAR pathway activation in Beas-2B cells upon PM2.5 exposure. (**A**,**B**) Beas-2B cells were transfected with ATR siRNA, CHK1 siRNA or their control siRNAs, respectively. Then the cells were exposed to PM2.5 (100 μg/mL) and the expression of TIGAR was examined at 12 h after PM2.5 exposure. (**C**,**D**) Beas-2B cells were treated with different doses of PM2.5 for 12 h or a single dose of PM2.5 (100 μg/mL) for the indicated time periods and then the activation of LKB1 was detected. (**E**) Beas-2B cells were transfected with LKB1 siRNA or control siRNA and then exposed to PM2.5 (100 μg/mL). The activation of p53/TIGAR pathway was examined at 12 h after PM2.5 exposure. (**F**) Beas-2B cells stably transfected with the p53-dependent luciferase reporter were transfected with LKB1 siRNA or control siRNA and treated with PM2.5 (100 μg/mL). The induction of the p53-dependent luciferase activity was determined at 12 h after PM2.5 exposure (**P < 0.01).

In the previous report, we have also revealed that ATR/CHK1 plays a critical role in mediating p53/DRAM1 pathway activation in the PM2.5-induced response^12^. Therefore in the following study, we focused on identifying the upstream protein kinase(s) that might be responsible for p53/TIGAR pathway activation in Beas-2B cells after PM2.5 treatment. To this end, we tested the activation status of several known p53 protein kinase (ATR, ATM, CHK1, LKB1, AMPKα, p38K) and their potential contribution to p53/TIGAR cascade activation. We found that ATR and CHK1, although functions in triggering p53/DRAM1 pathway activation, were not involved in TIGAR induction in PM2.5-treated Beas-2B cells (Fig. 3A,B). Similar response was also observed in the detection of ATM, AMPKα and p38K (data not shown). Interestingly, we observed both a dose- and time-dependent activation of LKB1 in Beas-2B cells after PM2.5 exposure (Fig. 3C,D). Most importantly, knocking down LKB1 expression completely blocked the phosphorylation and transactivation of p53 as well as TIGAR upregulation induced by PM2.5 (Fig. 3E,F). These data together indicate that LKB1 is required for p53/TIGAR pathway activation in Beas-2B cells upon PM2.5 exposure.

### LKB1 contributed to autophagy-dependent VEGF induction in Beas-2B cells upon PM2.5 exposure

**Figure 4:**
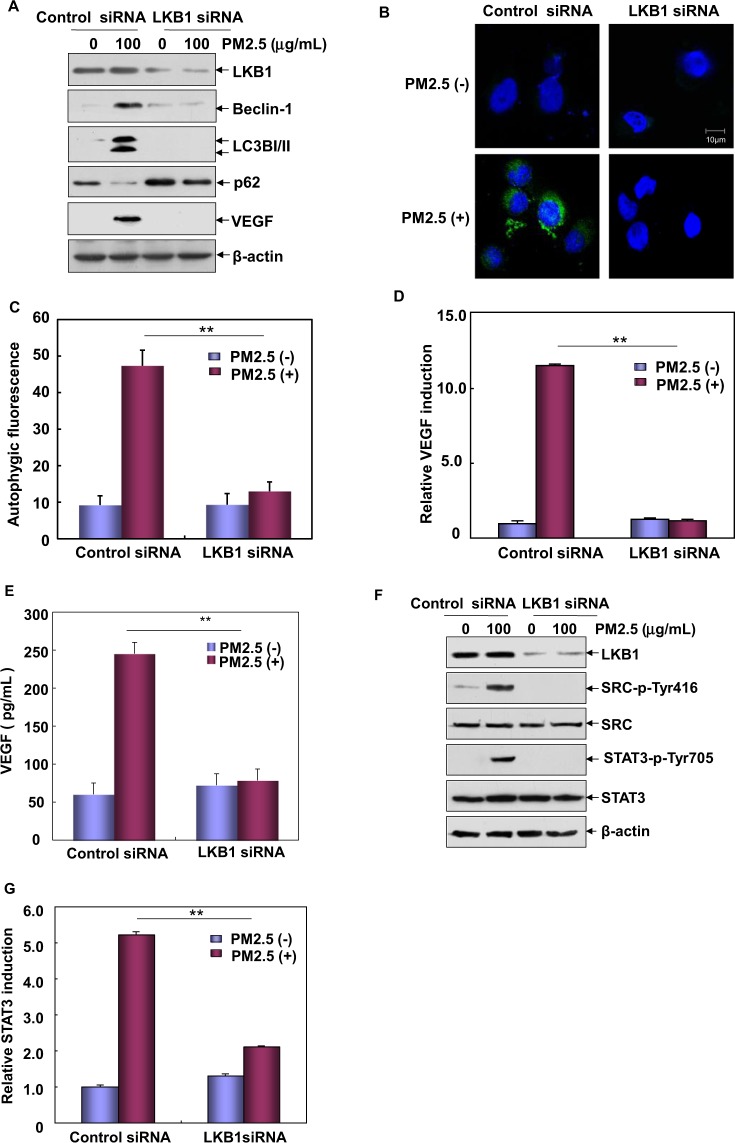
LKB1 contributed to autophagy-dependent VEGF induction in Beas-2B cells upon PM2.5 exposure. (**A**) Beas-2B cells were transfected with LKB1 siRNA or control siRNA and then exposed to PM2.5 (100 μg/mL). The expression levels of the autophagic hallmarks and VEGF were examined at 12 h after PM2.5 exposure. (**B**,**C**) Beas-2B cells were transfected and treated with PM2.5 (100 μg/mL) as described in (**A**). Then, the cells were stained with Cyto-ID Green Autophagy Detection Reagent, and the autophagy was examined by subjecting the cells to confocal microscopy assay or a flow cytometric analysis to quantitatively measure the autophagic fluorescence intensity inside the cells (^**^P < 0.01). (**D**) Beas-2B cells stably transfected with *VEGF* promoter-driven luciferase reporter were transfected with LKB1 siRNA or control siRNA and then treated with PM2.5 (100 μg/mL). The induction of *VEGF* promoter-dependent luciferase activity was determined at 12 h after PM2.5 exposure (**P < 0.01). (**E**) Beas-2B cells were transfected and treated with PM2.5 (100 μg/mL) as described in (**A**). Cell supernatants were collected and the expression of VEGF was examined by ELISA at 24 h after PM2.5 exposure (**P < 0.01). (**F**) Beas-2B cells were transfected and treated with PM2.5 (100 μg/mL) as described in (**A**). Then the activation of Src/STAT3 pathway was examined at 12 h after PM2.5 exposure. (**F**) Beas-2B cells stably transfected with the STAT3-dependent luciferase reporter were transfected with LKB1 siRNA or control siRNA and then treated with PM2.5 (100 μg/mL). The induction of the STAT3-dependent luciferase activity was determined at 12 h after PM2.5 exposure (**P < 0.01).

Next, we investigated whether LKB1 is also involved in regulating p53/TIGAR –dependent autophagy and VEGF induction in the Beas-2B cells under PM2.5 exposure. We observed that the upregulation of both LC3BI/II and Beclin1 and degradation of p62 induced by PM2.5 were efficiently inhibited by knockdown of LKB1 expression (Fig. 4A). Moreover, there was a significant reduction in the specific autophagic fluorescence signals from the Cyto-ID Autophagy Detection Reagent-stained Beas-2B cells with the impairment of LKB1 expression (Fig. 4B,C). Under the same conditions, both the transcription and the protein syntheses of VEGF were totally blocked (Fig. 4A,D,E). These data indicate that LKB1 is also required for autophagy and VEGF induction in Beas-2B cells upon PM2.5 exposure. In the following study, we further observed that knocking down LKB1 expression significantly inhibited that Src/STAT3 pathway activation in Beas-2B cells (Fig. 4F,G). Taken these data together, we conclude that PM2.5 exposure can activate LKB1/p53/TIGAR pathway, leading to autophagy-dependent VEGF expression by linking Src/STAT3 signaling cascade.

### PM2.5 exposure resulted in elevated concentration of airway VEGF and triggered the inflammatory responses in the pulmonary vascular endothelium

**Figure 5:**
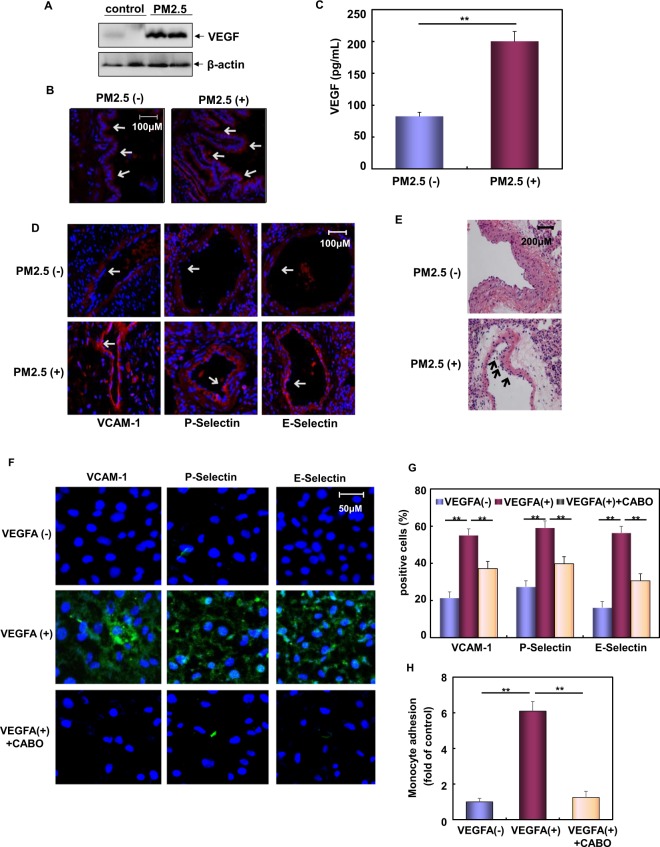
PM2.5 exposure resulted in elevated concentration of airway VEGF and triggered the inflammatory responses in the pulmonary vascular endothelium. (**A**) SD rats were left untreated or exposed to PM2.5 (3 mg/kg body weight) and then the lung tissue extracts were prepared. Then the pulmonary VEGF expression was detected by western-blot assay. (**B**) The lung tissue sections from SD rats untreated or exposed to PM2.5 were prepared and *in situ* VEGF expression in the bronchial epithelium was detected by immunofluorescence assay. The specific signal for VEGF expression (red fluorescence) was indicated as arrows. (**C**) The bronchial alveolar lavage fluid was collected from SD rats untreated or exposed to PM2.5. Then VEGF concentration was detected by ELISAs. (**D**) The lung tissue sections from SD rats untreated or exposed to PM2.5 were used to detect the expressions of adhesion molecules driving vascular inflammation in pulmonary vascular endothelium by immunofluorescence assay. The specific signals for VCAM-1, E-Selectin and P-Selectin expression (red fluorescence) were indicated as arrows. (**E**) The inflammatory response in the pulmonary vascular endothelium was detected by HE staining. The arrows indicated monocyte infiltration on the vascular endothelium of rats exposed to PM2.5. (**F**,**G**) HUVECs were left untreated or treated with VEGFA (5 ng/mL) for 24 h with or without pretreatment of cabozantinib (1 nM) for 2 h. Then surface level of VCAM-1, E-Selectin and P-Selectin was detected by immunofluorescence assay and the quantitative flow cytometric assay (**P < 0.01). (**H**) HUVECs in (**F**) and (**G**) were co-cultured with BCECF/AM-labeled U937 cells. The adhesion of U937 cells to the vascular endothelial cells were evaluated by quantitative flow cytometric assay (**P < 0.01).

In order to further confirm the pathological role of VEGF induction in mediating the pulmonary inflammatory responses, *in vivo* analysis was then performed in the SD rats exposed to Wuhan PM2.5. Because we have demonstrated that PM2.5 can be directly taken up into Beas-2B cells in our previous report^12^, rats were intratracheally (IT) instilled with PM2.5 to facilitate the absorption of PM2.5 by airway epithelial cells. As shown in Fig. 5A, inhalation of PM2.5 led to a strong induction of VEGF expression in the pulmonary tissue extract, accompanying with significant upregulation of VEGF expression in the bronchial epithelium of PM2.5-treated rats and elevation of VEGF concentration in the bronchial alveolar lavage fluid (Fig. 5B,C). These results indicate that PM2.5 exposure is able to induce airway VEGF upregulation in rats.

To determine whether VEGF induction can trigger a vascular inflamed response, we next examined the expressions of adhesion molecules driving vascular inflammation in pulmonary vascular endothelium of rats treated with PM2.5. The results from immunofluorescence assays showed higher level of VCAM-1, E-Selectin and P-Selectin expressions on the surface of vascular endothelium after PM2.5 treatment (Fig. 5D). Under the same conditions, a significant increase in HE staining signal indicating the infiltration of monocyte on the vascular endothelium was readily observed upon PM2.5 exposure (Fig. 5E). These data indicate that PM2.5 exposure also induces pulmonary vascular endothelium dysfunction along with airway VEGF upregulation.

To determine the potential functional link between VEGF upregulation and the vascular endothelium dysfunction under PM2.5 exposure, an *in vitro* assay was performed in human umbilical vein endothelial cells (HUVECs) under the treatment of VEGFA to mimic the local response within the bronchial tube and its surrounding vessels under VEGF upregulation. Here we found that VEGFA exposure resulted in a significant upregulation of VCAM-1, E-Selectin and P-Selectin expressions in HUVECs, which responses were efficiently interrupted by pre-treatment of the specific VEGFRII inhibitor, Cabozantinib (Fig. 5F,G). Under the same conditions, VEGFA-induced adhesion of U937 monocytes to HUVECs was nearly total blocked under Cabozantinib pre-treatment (Fig. 5H). These data together indicate that VEGF induction efficiently irritates inflammatory responses on the vascular endothelium.

## Discussion

The induction of pulmonary inflammation is regarded as a critical mechanism for PM2.5-induced adverse effects in the cardiopulmonary systems^1,2^. Therefore, illustrating the signaling events in mediating the airway and vascular inflammatory responses is the major issue in the toxicological research of particle. In our recent report, we have demonstrated that PM2.5 exposure induces local upregulation of VEGF expression in the airway by activating p53-dependent autophagy^12^. Therefore, in the current study, we conducted a further-in-depth research to investigate the upstream and downstream signaling events relating to p53/autophagy axis activation and VEGF induction in the PM2.5-treated bronchial epithelial cells and the biological significance of airway VEGF upregulation in the PM2.5-treated rats.

One of the important findings in this study was that LKB1 acted as the upstream protein kinase responsible for p53-dependent autophagy induction. As a serine/threonine protein kinase, LKB1 not only functions as a well-established tumor suppressor^15,16^, but also acts as a master upstream kinase of AMPKs, the key regulator for glucose and lipid metabolism upon diverse stimuli. Therefore, activation of LKB1/AMPK axis is involved in a wide range of cellular functions^17^. Induction of autophagy has been reported to be a major event after LKB1/AMPK pathway activation under a variety of stress conditions^18^. This response is mediated by the negative regulation of AMPK on mTORC1 activity, followed by relieve of the mTOR inhibition on autophagy induction^19–22^. Recently, a LKB1-dependent, while AMPK/mTOR-independent autophagy was also observed in the lung cancer cells^23^, indicating that LKB1 might link to autophagy beyond AMPK and mTOR-related events. In this study, we discovered that LKB1 activation induced the autophagic flux in the PM2.5-treated human bronchial epithelial cells by triggering p53 phosphorylation and TIGAR upregulation. These results not only provided novel discoveries in the functional mechanism of LKB1-dependent autophagy, but also identified a new regulator for p53/autophagy axis under stress conditions.

The role of LKB1 involving in regulating the inflammation has been widely investigated in the previous reports^24–31^. Most of the data supports that LKB1 acts as an anti-inflammatory effector. It can not only exert the protective functions in antagonizing the inflammatory responses induced by mycobacterial infection, LPS or FGF21^24,27,29^, but also play critical roles in controlling the inflammation in T cells, cardiomyocyte, renal epithelial cilia, brown adipose tissue, skeleton muscle, etc.^25,26,28,30,31^. In the current study, we have provided the novel evidence that LKB1 was involved in mediating the proinflammatory responses in the bronchial epithelial cells upon PM2.5 exposure. Combined with a previous report in which LKB1 was shown to be required for thromboxane receptor-dependent NF-κB activation and proinflammatory cytokines induction in the HUVECs^32^, we believe that the regulatory role of LKB1 in inflammatory responses may be different under various conditions.

TIGAR was initially identified as a transcriptional target of p53 with the function in controlling the metabolism^14^. However, p53-independent expression of TIGAR has also been detected in several human cancer cell lines^33^, although the according mechanism involving p53-independent regulation of TIGAR is little known. In fact, TIGAR is a multi-functional protein, which is involved in regulating not only metabolism, but also apoptosis, autophagy, cell cycle progression and radiation response^34^. The role and mechanism of p53/TIGAR axis in attenuating mitophagy to exacerbate cardiac damage after ischemia has been linked to the antioxidant activity of TIGAR in lowing ROS accumulation^35^. And the p53-independent TIGAR expression is also shown to limit autophagy under nutrient deprivation or metabolic stress by restraining ROS levels^36^. In the current study, we have identified the opposite role of TIGAR in promoting autophagy in the bronchial epithelial cells upon PM2.5 exposure, which response was strictly depended on the transactivity of p53. Therefore, we conclude that p53/TIGAR axis activation may positively or negatively regulate autophagy under different stress conditions. More importantly, both p53-dependent and independent TIGAR expression have been shown to modulate autophagy and subsequent apoptosis according to the previous reports^14,35,36^. Data in the current study have revealed the contribution of p53/TIGAR axis activation to the induction of VEGF expression and airway inflammatory responses, which provided the expanding discovery for the functional research of this important cascade.

In conclusion, we have identified LKB1/p53/TIGAR- dependent autophagy in mediating VEGF production and pulmonary inflammatory responses induced by PM2.5. Alleviating autophagy induction or suppressing VEGF expression in the airway might be helpful for overcoming PM2.5-induced cardiopulmonary injuries.

## Methods

### Antibodies, siRNAs and reagents

The following primary antibodies were used in this study, including Beclin1 (CST, 3495), LC3BI/II (CST, 3868), p62 (CST, 8025), p53-p-Ser15(CST, 9284), p53 (CST, 2524), Src-p-Tyr416 (CST, 6943), Src (CST, 2109), STAT3-p-Tyr705 (CST, 9145), STAT3 (CST, 9139), LKB1-p-Ser28 (CST, 3482), LKB1 (CST, 3047), ATR (CST, 2790), CHK1 (CST, 2360), VEGF(sc-65617), TIGAR (sc-166291), Sestrin2 (CST, 8487), DAPK1 (CST, 3008), VCAM-1(SC-13160), P-Selectin (SC-8419), E-Selectin (SC-71017), and β-actin (sc-69879) antibodies. The siRNAs specific targeting TIGAR, Sestrin2, DAPK1, ATR, CHK1, LKB1, p53, and their control siRNAs were purchased from Riobo Technology (Guangzhou, China). VEGFA was purchased from Proteintech Group. Inc (IL, USA). The VEGFR II inhibitor cabozantinib was purchased from MedChemExpress (NJ, USA). The Cyto-ID Autophagy Detection Kit was purchased from ENZO Biochem (NY, USA)

### Cell culture

Beas-2B human bronchial epithelial cells and its stable transfectants (Beas-2B-*VEGF*-Luc, Beas-2B-p53-Luc and Beas-2B-STAT3-Luc) were established and described in our previous report^12^. Human umbilical vein endothelial cells (HUVECs) and human monocytic leukemia cells (U937) were described in our previous study^37^. The cells were maintained in DMEM (Life Technologies) with 10% fetal bovine serum (Life Technologies) supplemented with antibiotic/antimycotic (Life Technologies).

### PM2.5 samples collection, preparation and cell/animal treatments

PM2.5 sample used in this study was collected in the Hanyang District of Wuhan, China. To keep the filter samples stable, a constant temperature and relative humidity (−20 ± 0.5 °C, 40 ± 5% RH) were used to store the filters. To prepare the PM2.5 samples, particle was extracted from the water-immersed filters by sonicating and then the water-extracted samples were frozen to powder. The powder were weighed, sub-packed and stored at −20 °C until use. For cell treatment, the PM2.5 sample powder was suspended in cell culture medium, and diluted to the final concentrations after sonication for 5 min. Contamination of endotoxin in PM2.5 samples has been excluded according to our previous results^11^. Animal treatment was performed as described previously^37^. The protocols of all animal experiments were approved by the Ethics Committee of Institute of Military Cognitive and Brain Sciences. All methods were performed in accordance with the relevant guidelines and regulations.

### RT-PCR assay

Total RNA extraction and the reverse transcription were performed as described previously^12,37^. The following oligonucleotides were synthesized and used to analyze the induction of *TIGAR, Sestrin2* and *DAPK1* transcription: *TIGAR*: forward: 5′-GCTGCCGATGTCAGAATGGT-3′), reverse:(5′-TTCACCTCGAGTG ACTTGCC-3′); *Sestrin2*: forward: 5′-ACTCAGCGAGATCAACAAGT-3′), reverse: (5′-TCTGTTCACTAGGGGGTGTA-3′); *DAPK1*: forward: 5′-CGTACCCTCCTGGATTGTGG-3′), reverse: (5′-TCCTGTTGTCACA GAAGGGC-3′).

### HE staining

Lung tissue from the SD rats untreated or exposed to PM2.5 (3 mg/kg body weight) were immersed in 4% polyformaldehyde and the paraffin section samples were prepared by conventional method. Before immunostaining, the lung tissue sections were dewaxed in the xylene, rehydrated through decreasing concentrations of ethanol, and then stained with hematoxylin and eosin (H&E). After staining, the sections were dehydrated through increasing concentration of ethanol and xylene.

### Monocytic adhesion assay

U937 cells were labeled with BCECF/AM (Life Technologies) following the instruction of the manufacture. Then the labeled U937 cells were added into the VEGFA-treated or untreated HUVECs for co-culture. The adhesion of U937 cells to the vascular endothelial cells were evaluated quantitatively by detecting the fluorescence intensity with flow cytometric assay (BD Biosciences, FACSCalibur).

Other experimental methods, including Western-blot assay, Luciferase reporter assay, Autophagy assay, Immunofluorescence assay, ELISAs, Cell variability assay and the Statistical analysis were performed as described in our previous reports^12,37^.

## Supplementary information

Supplementary Information
