# Supplementary material for: LKB1/p53/TIGAR/autophagy-dependent VEGF expression contributes to PM2.5-induced pulmonary inflammatory responses
Source: Sci Rep. 2019 Nov 12;9:16600. doi: 10.1038/s41598-019-53247-6 (PMC6851103; doi:10.1038/s41598-019-53247-6)
Supplement: Supplementary file 1 — Supplementary Information [file 41598_2019_53247_MOESM1_ESM.pdf]

LKB1/p53/TIGAR/autophagy-dependent VEGF expression contributes to PM2.5-induced pulmonary inflammatory responses

Huan Xu[1, 2], Xiuduan Xu[1, 2, #], Hongli Wang[1, 3], Aodeng Qimuge[1, 4], Shasha Liu[1, 5], Yuanlian Chen[1, 6], Chongchong Zhang[1, 3], Meiru Hu[1], Lun Song[*, 1, 2]

Supplementary information

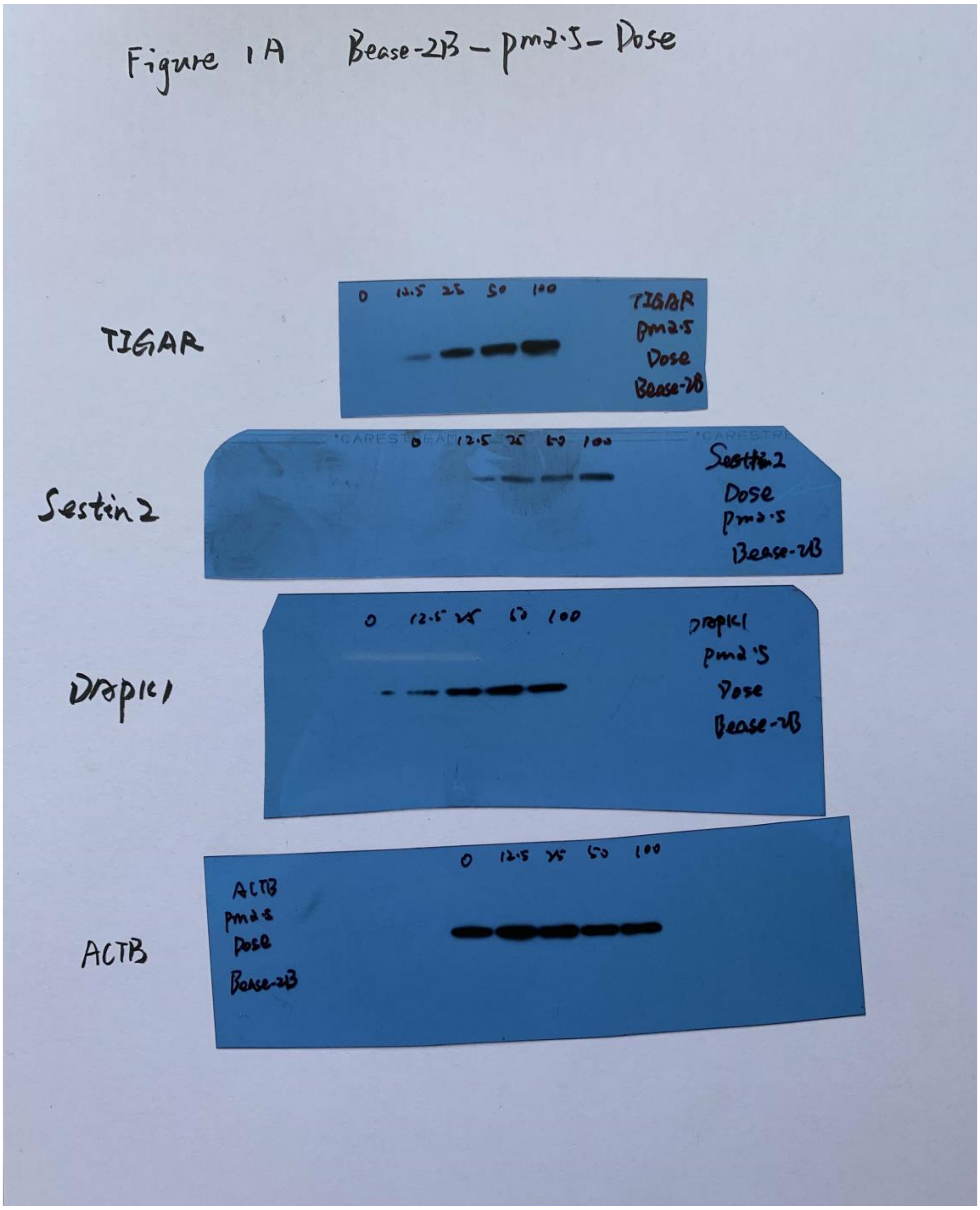

Figure 1A Bease-2B – pm2.5 – Dose
TIGAR
0 12.5 25 50 100
TIGAR
pm2.5
Dose
Bease-2B
Sestin2
0 12.5 25 50 100
Sestin2
Dose
pm2.5
Bease-2B
0 12.5 25 50 100
pm2.5
Dose
Bease-2B
ACTB
0 12.5 25 50 100
ACTB
pm2.5
Dose
Bease-2B

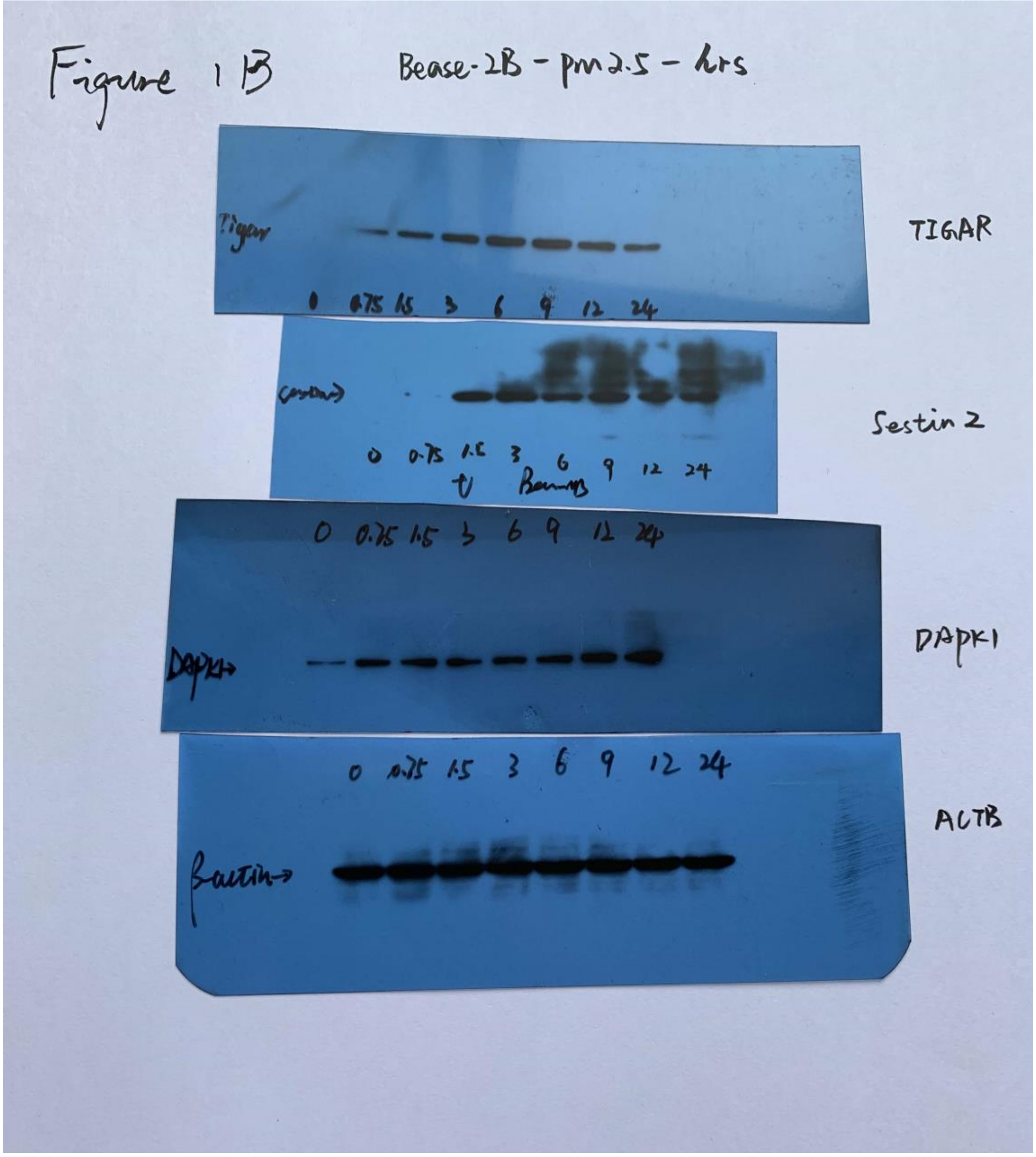

Figure 1B
Bease-2B - pm2.5 - hrs
TIGAR
0 0.75 1.5 3 6 9 12 24
Sestin 2
0 0.75 1.5 3 6 9 12 24
0 0.75 1.5 3 6 9 12 24
DAPK1
0 0.75 1.5 3 6 9 12 24
ACTB

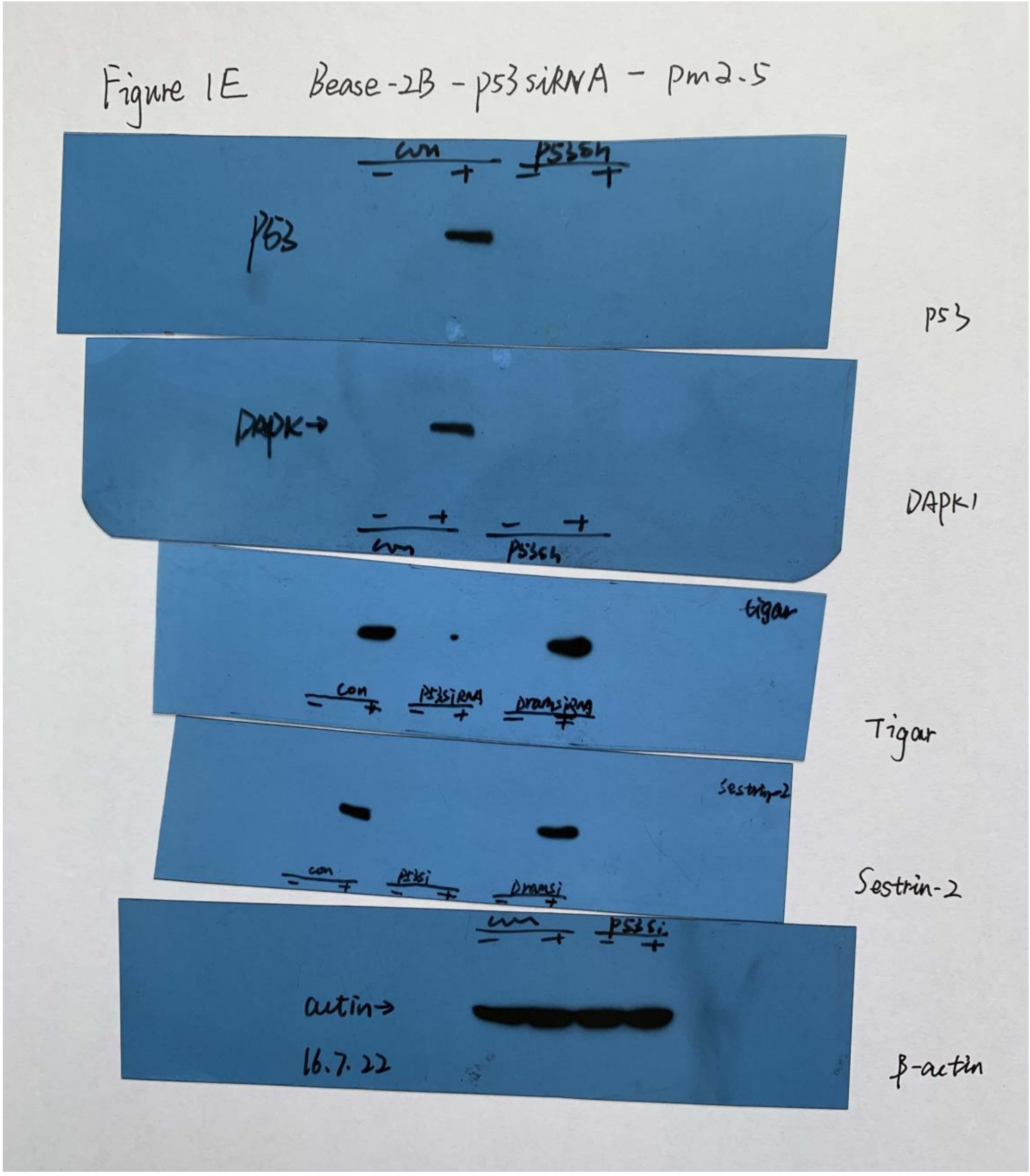
Figure 1E Bease-2B - p53siRNA - pm2.5
p53
DAPK→
DAPK1
Tigar
Sestrin-2
actin→
16.7.22
β-actin

Figure 2A Bease-2B - Sestrin2 siRNA - pm2.5

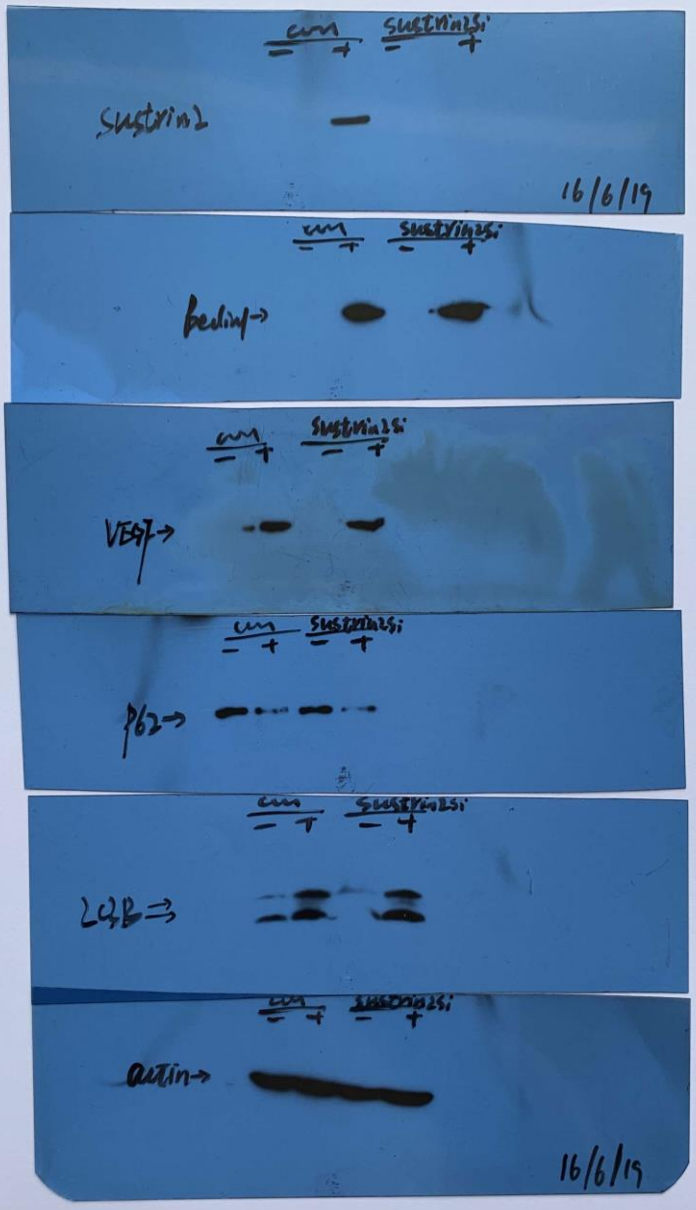

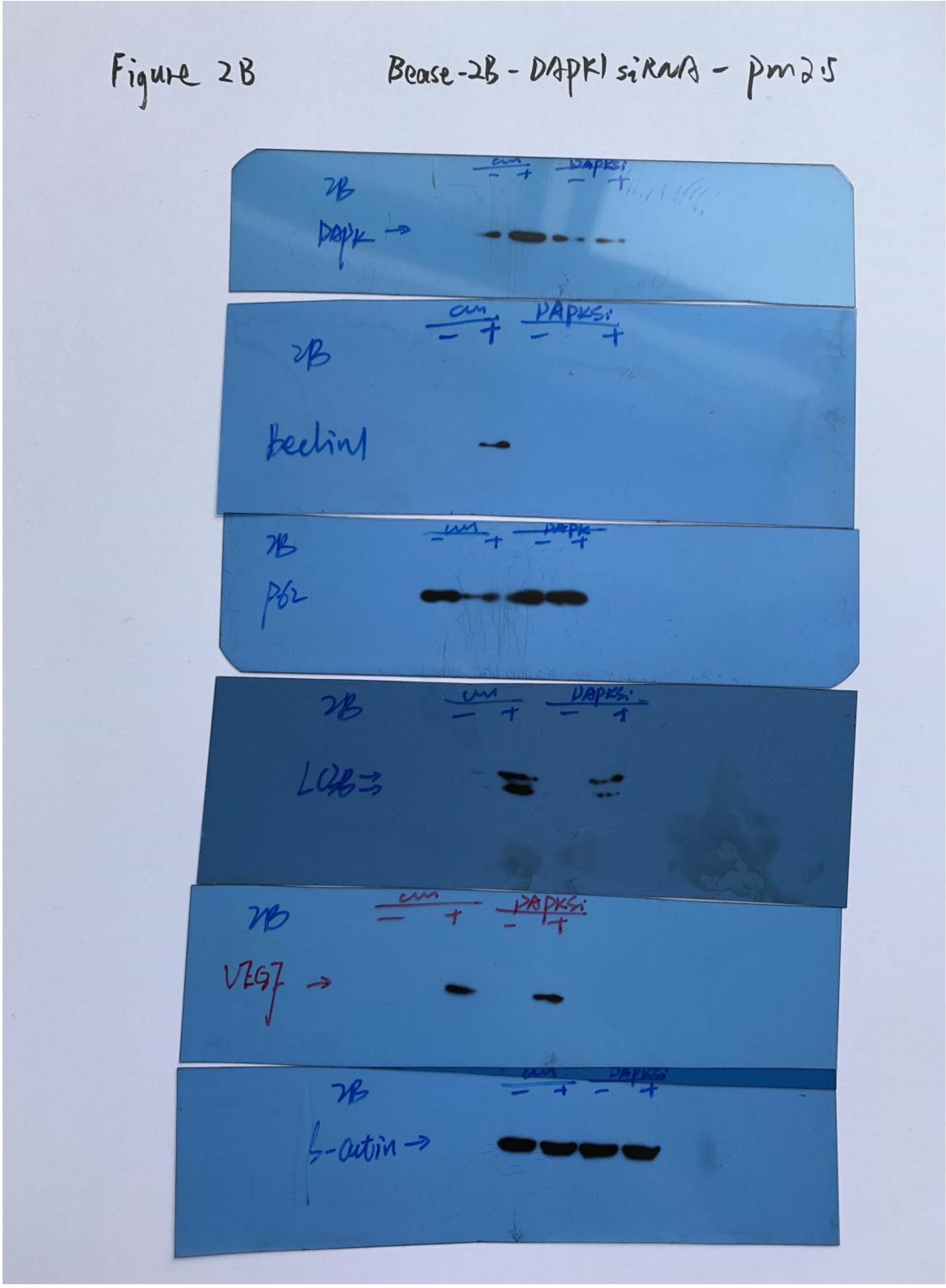
Figure 2B
Bease-2B - DAPK1 siRNA - pm2.5
DAPK →
beclin1
P62
LC3B
VEGF →
β-actin →

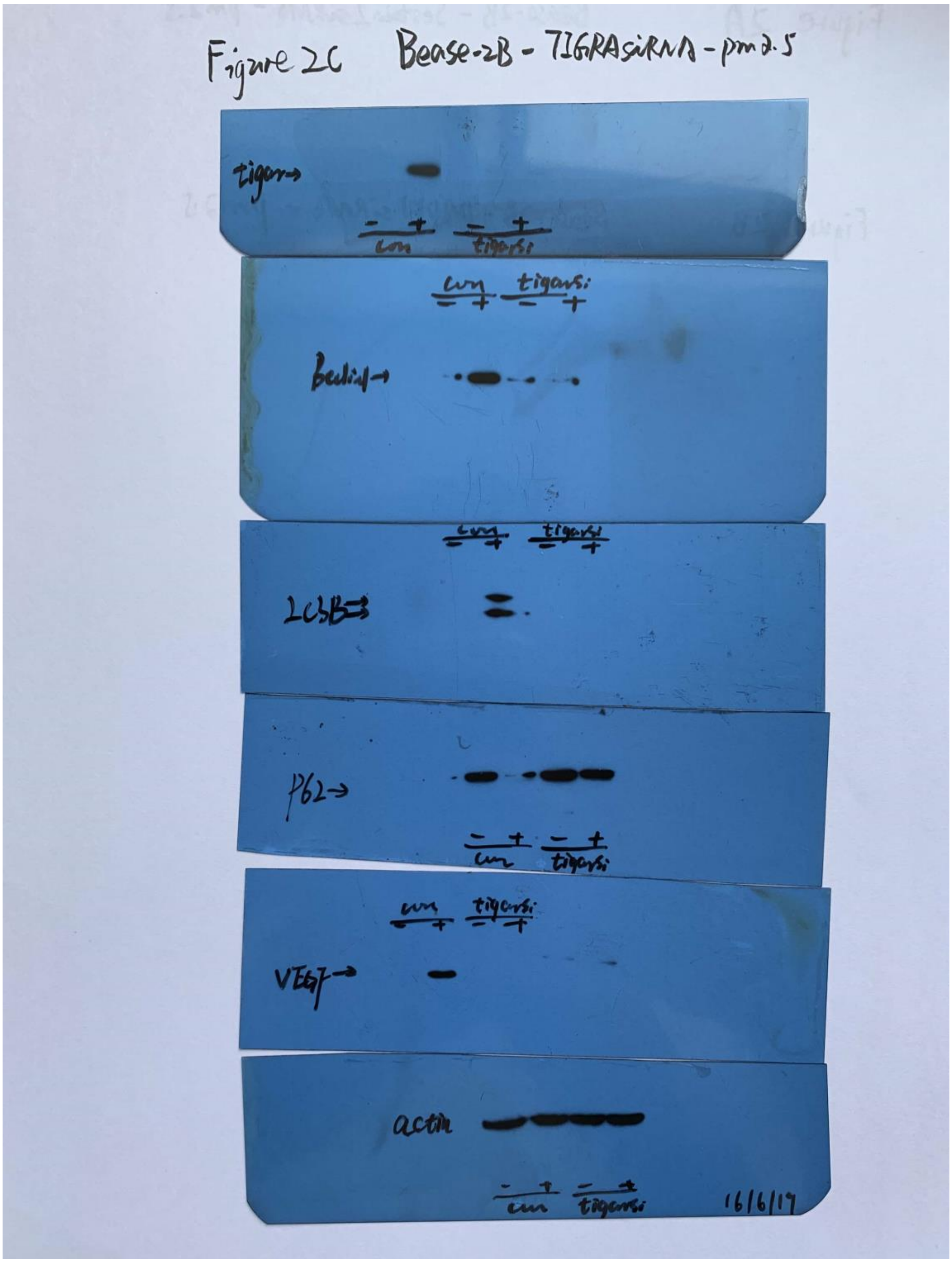
Figure 2C Bease-2B - TIGRAsiRNA - pm2.5
tiger→
− + − +
con tigarsi
Beclin→
LC3B⇉
P62→
VEGF→
actin
16/6/19

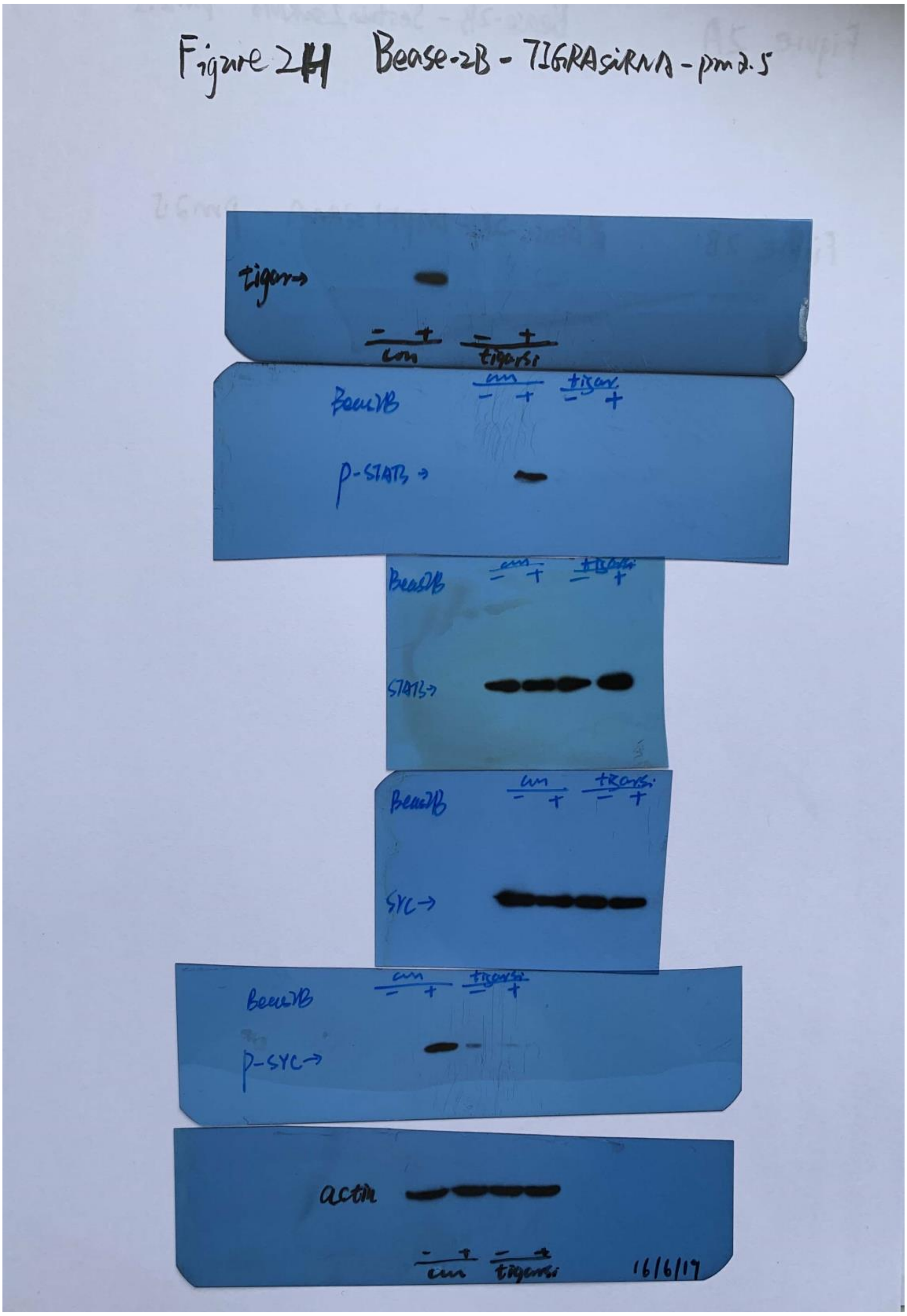

Figure 2H Bease-2B - TIGRAsiRNA - pm2.5
tiger→
- + - +
con tigarsi
Beas2B
con tigar
- + - +
p-STAT3 →
STAT3→
SYC→
p-SYC→
actin
16/6/17

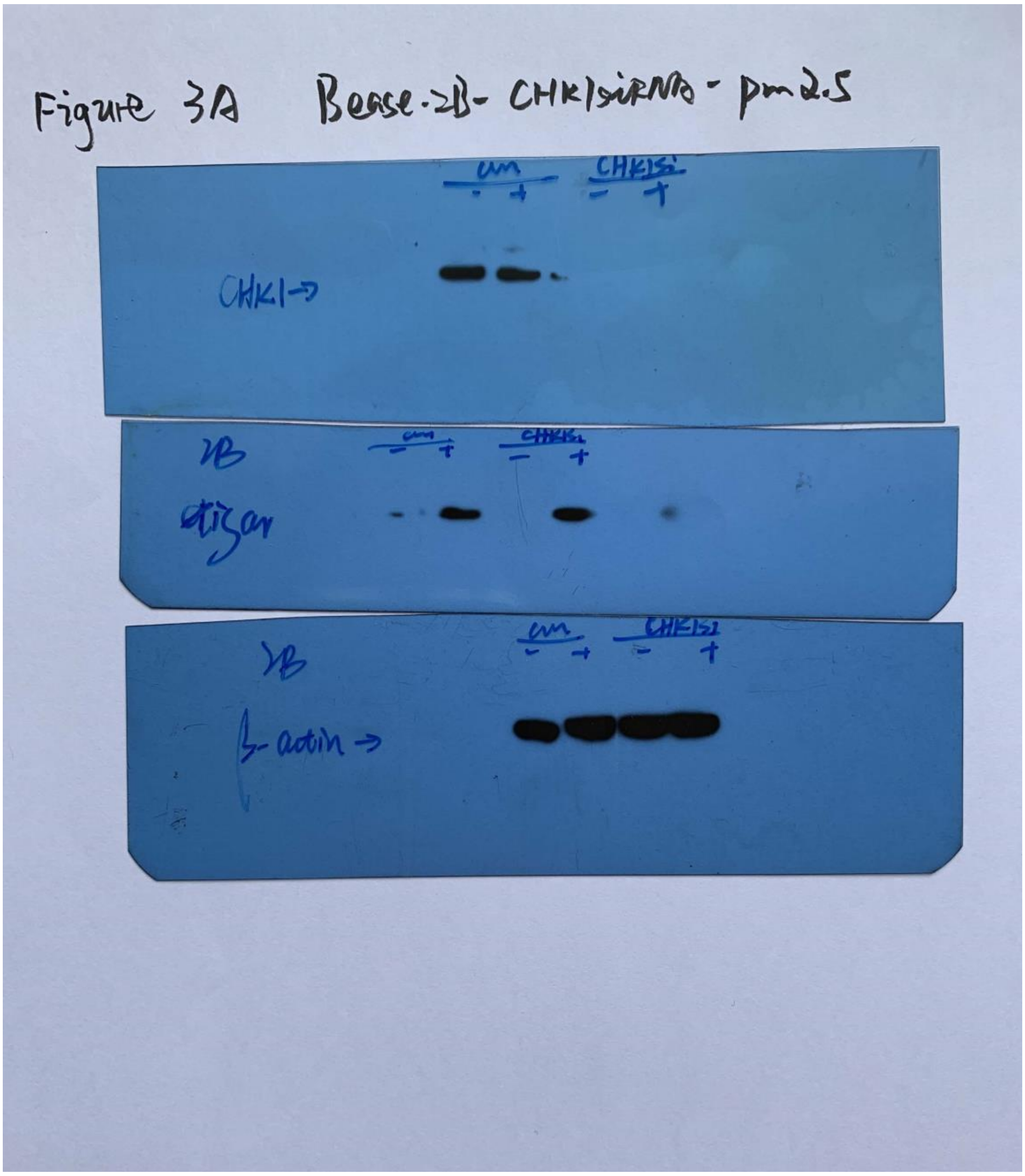
Figure 3A Beas-2B- CHK1siRNA - pm2.5
con
CHK1si
- +
- +
CHK1 →
2B
β-actin →

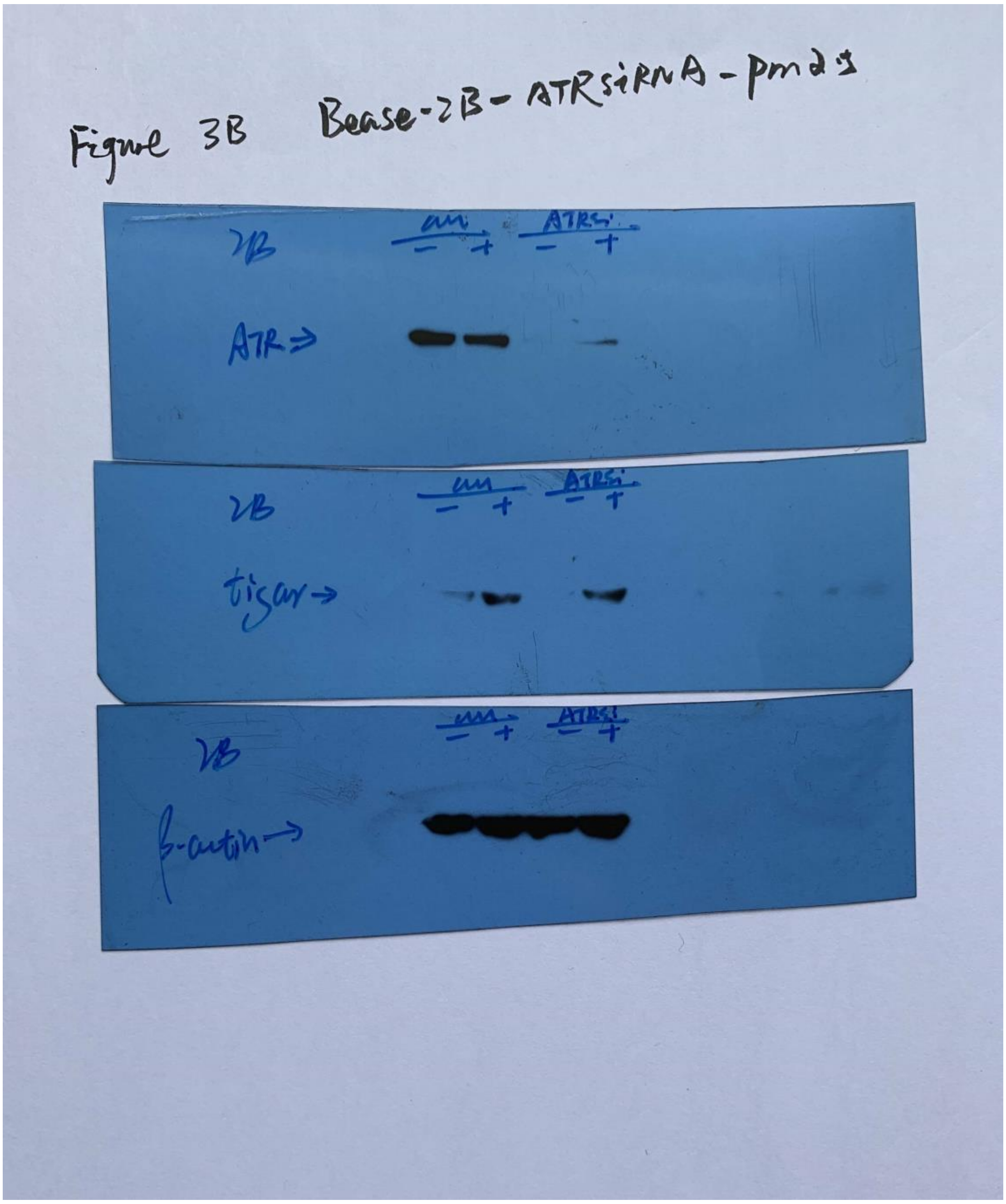
Figure 3B Bease-2B - ATRsiRNA - pm2.5
2B
ATRsi
− + − +
ATR ⇒
2B
ATRsi
− + − +
tigar →
2B
ATRsi
− + − +
β-actin →

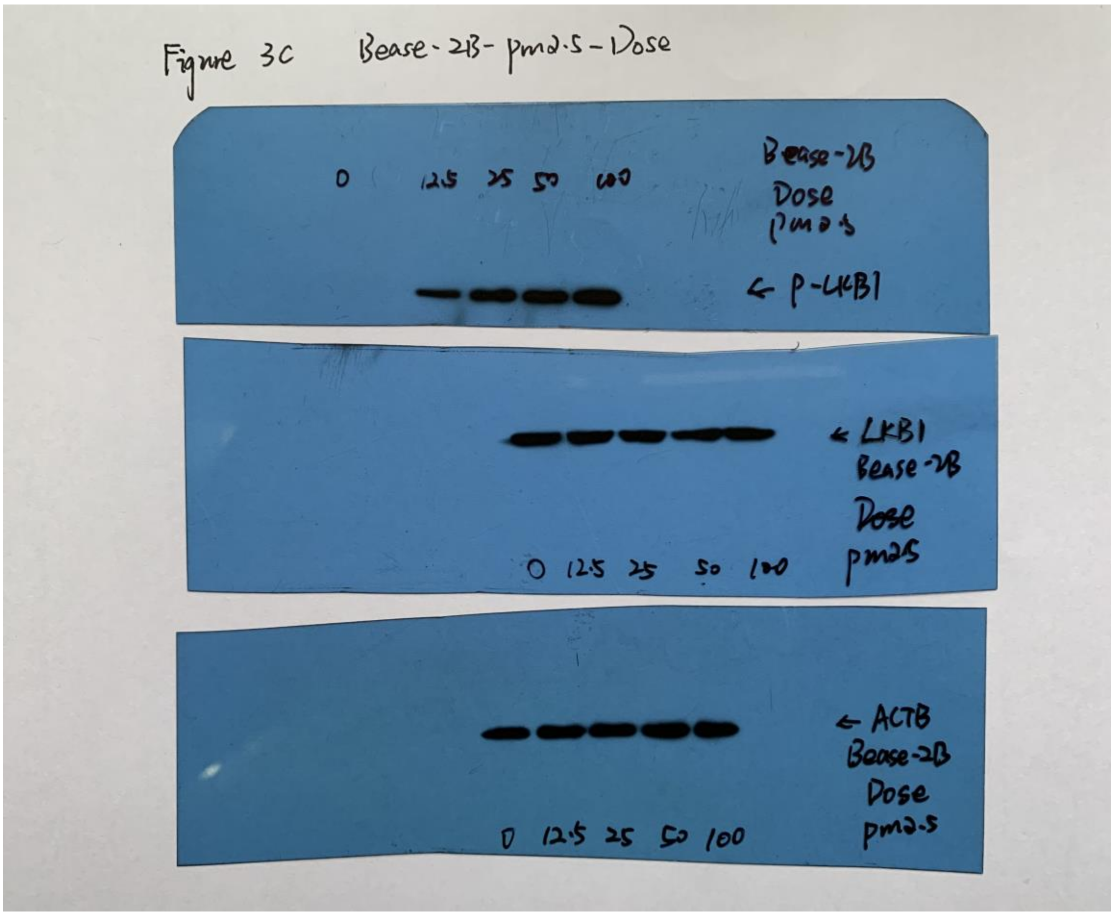
Figure 3C Bease-2B-pm2.5-Dose
0 12.5 25 50 100
Bease-2B
Dose
pm2.5
← p-LKB1
← LKB1
Bease-2B
Dose
pm2.5
0 12.5 25 50 100
← ACTB
Bease-2B
Dose
pm2.5
0 12.5 25 50 100

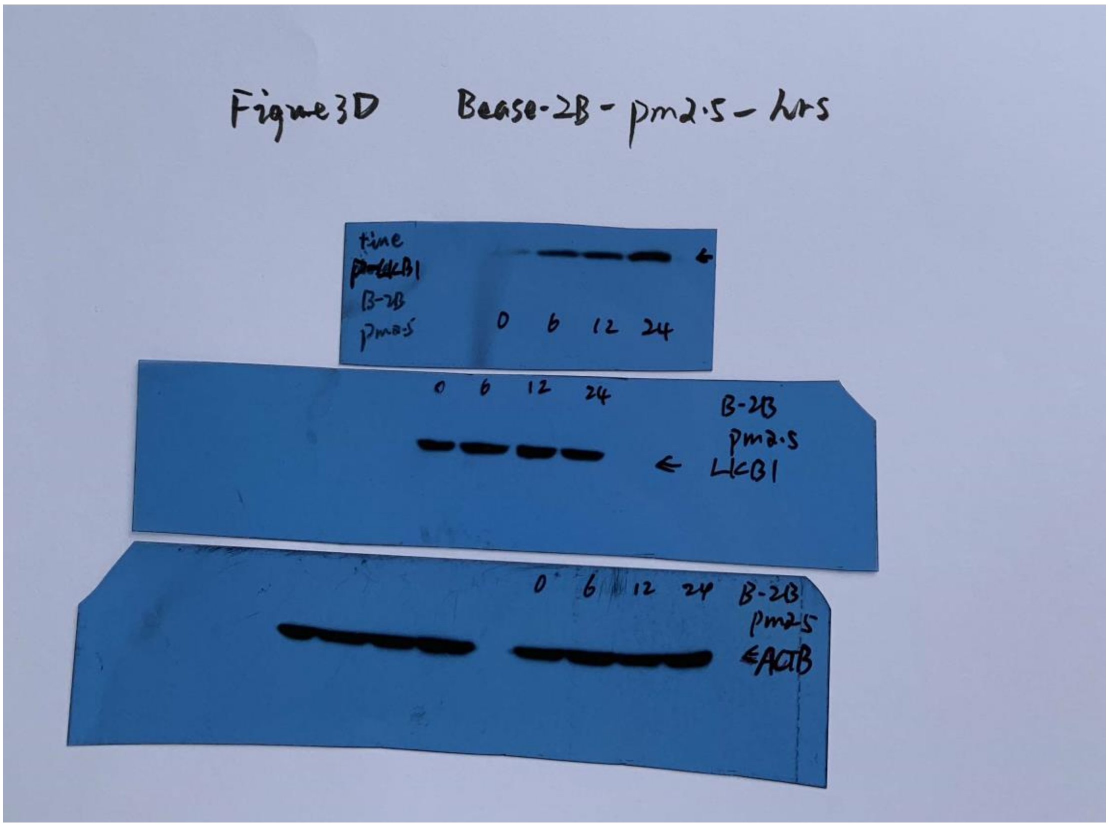
Figure3D Bease-2B - pm2.5 - hrs
time
p-LKB1
B-2B
pm2.5
0 6 12 24
0 6 12 24
B-2B
pm2.5
LKB1
0 6 12 24
B-2B
pm2.5
ACTB

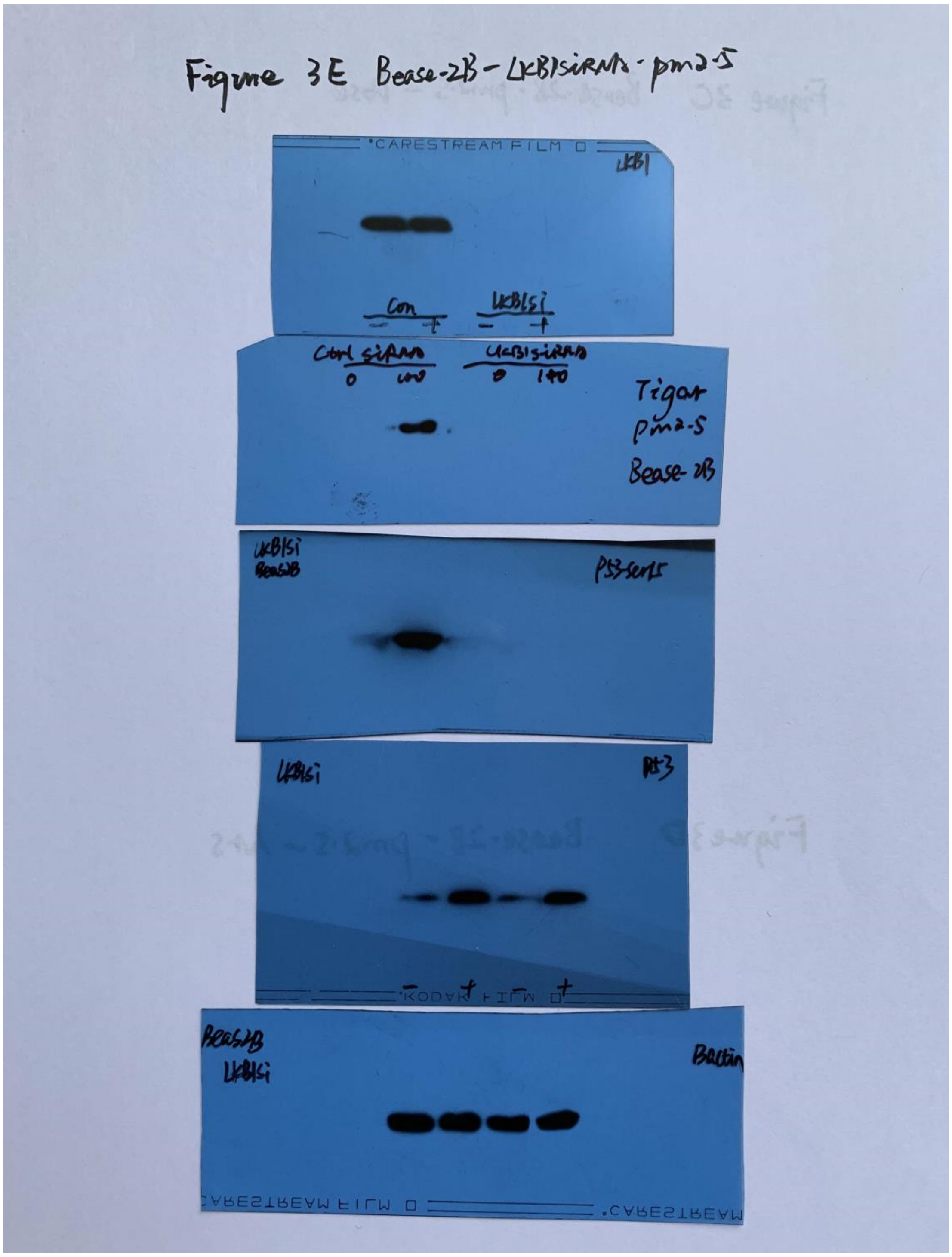
Figure 3E Bease-2B – LKB1siRNA - pm2.5
CARESTREAM FILM
LKB1
Con
LKB1si
– +
– +
Ctrl siRNA
LKB1siRNA
0 100
0 100
Tigar
pm2.5
Bease-2B
LKB1si
Beas2B
P53-ser15
LKB1si
P53
– + – +
KODAK FILM
Beas2B
LKB1si
Bactin
CARESTREAM FILM
CARESTREAM

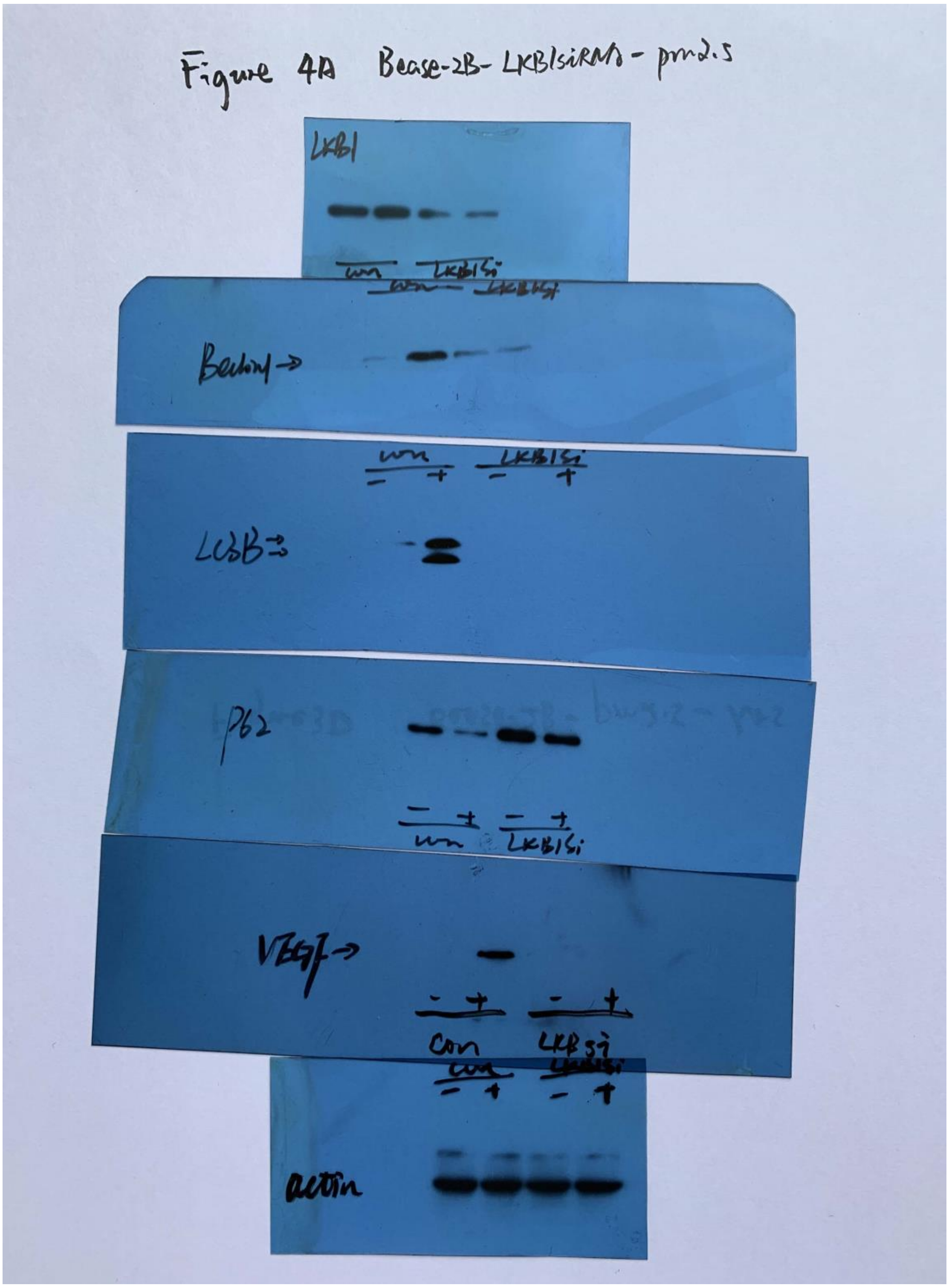
Figure 4A Bease-2B-LKB1siRNA-pm2.5
LKB1
con LKB1si
Beclin1
con LKB1si
- + - +
LC3B
P62
- + - +
con LKB1si
VEGF
- + - +
con LKB si
con LKB1si
- + - +
actin

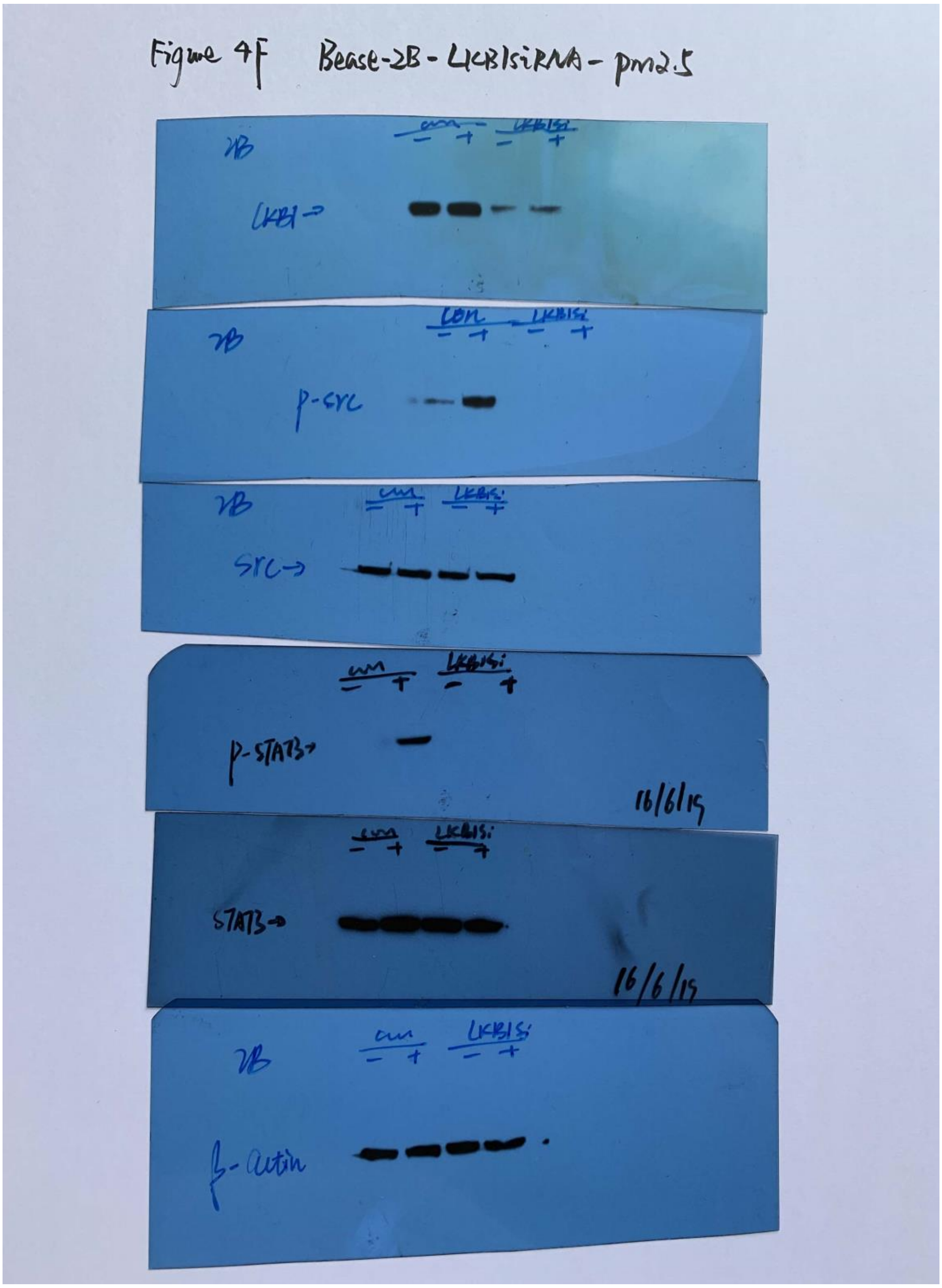
Figure 4F Bease-2B - LKB1siRNA - pm2.5
2B
con
LKB1si
− + − +
LKB1 →
p-src
src →
p-STAT3 →
16/6/19
STAT3 →
β-actin

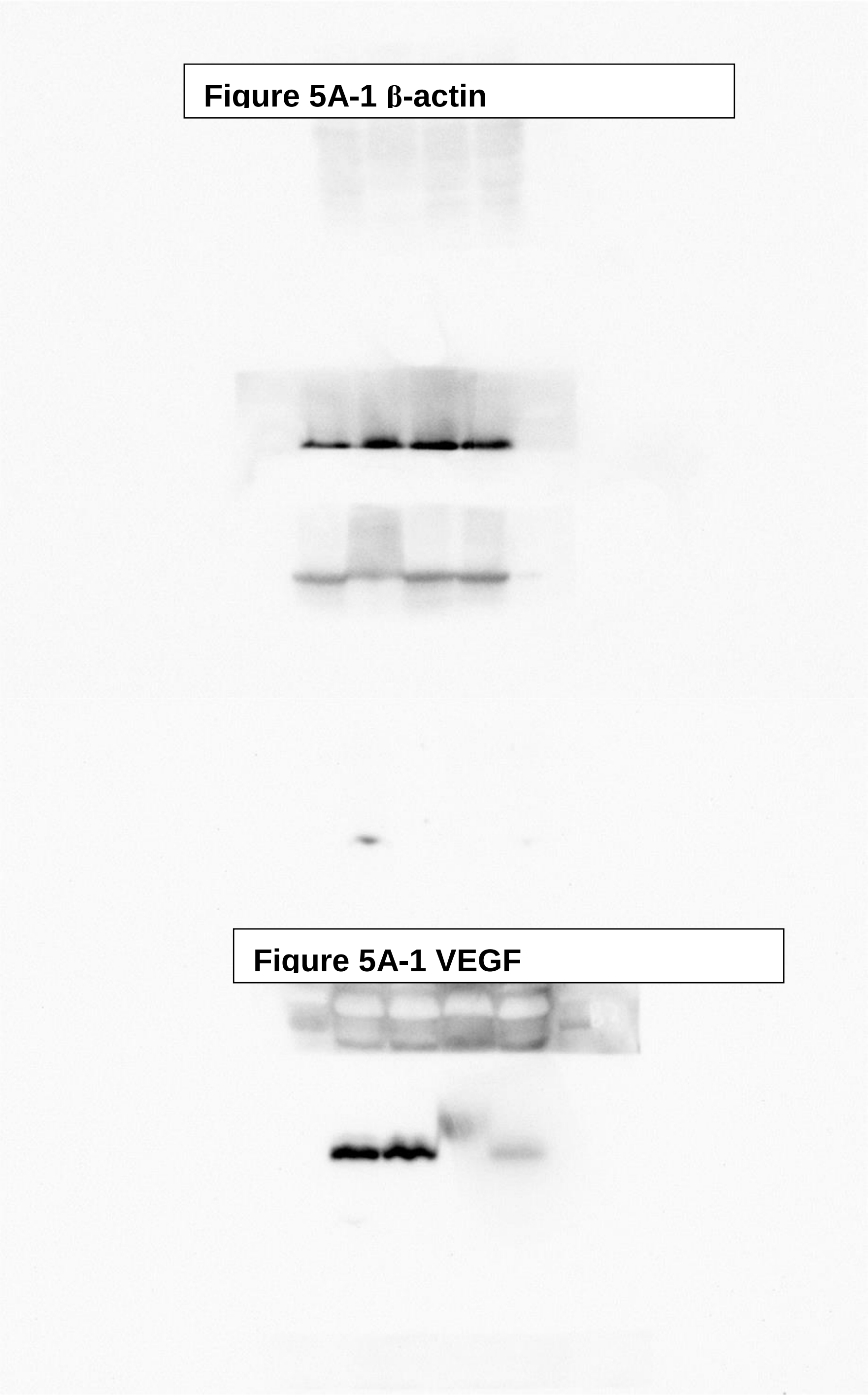
Figure 5A-1 β-actin
Figure 5A-1 VEGF
